# Atorvastatin reduces T-cell activation and exhaustion among HIV-infected cART-treated suboptimal immune responders in Uganda: a randomised crossover placebo-controlled trial

**DOI:** 10.1111/tmi.12442

**Published:** 2015-01-06

**Authors:** Damalie Nakanjako, Isaac Ssinabulya, Rose Nabatanzi, Lois Bayigga, Agnes Kiragga, Moses Joloba, Pontiano Kaleebu, Andrew D. Kambugu, Moses R. Kamya, Rafick Sekaly, Alison Elliott, Harriet Mayanja-Kizza

**Affiliations:** 1 Department of Medicine, Makerere University College of Health Sciences, Kampala, Uganda; 2 Infectious Diseases Institute, Makerere University College of Health Sciences, Kampala, Uganda; 3 Department of Medical Microbiology, Makerere University College of Health Sciences, Kampala, Uganda; 4 Medical Research Council Uganda/Uganda Virus Research Institute, Entebbe, Uganda; 5 Vaccine and Gene Therapy Institute of Florida, Port Saint Lucie, FL, USA

**Keywords:** atorvastatin, HIV/AIDS, antiretroviral therapy, adjunct therapy, immune activation, immune recovery

## Abstract

**OBJECTIVE:**

T-cell activation independently predicts mortality, poor immune recovery and non-AIDS illnesses during combination antiretroviral therapy (cART). Atorvastatin showed anti-immune activation effects among HIV-infected cART-naïve individuals. We investigated whether adjunct atorvastatin therapy reduces T-cell activation among cART-treated adults with suboptimal immune recovery.

**METHODS:**

A randomised double-blind placebo-controlled crossover trial, of atorvastatin 80 mg daily *vs.* placebo for 12 weeks, was conducted among individuals with CD4 increase <295 cells/μl after seven years of suppressive cART. Change in T-cell activation (CD3 + CD4 + /CD8 + CD38 + HLADR+) and in T-cell exhaustion (CD3 + CD4 + /CD8 + PD1 + ) was measured using flow cytometry.

**RESULTS:**

Thirty patients were randomised, 15 to each arm. Atorvastatin resulted in a 28% greater reduction in CD4 T-cell activation (60% reduction) than placebo (32% reduction); *P* = 0.001. Atorvastatin also resulted in a 35% greater reduction in CD8-T-cell activation than placebo (49% *vs.* 14%, *P* = 0.0009), CD4 T-cell exhaustion (27% *vs.* 17% in placebo), *P* = 0.001 and CD8 T-cell exhaustion (27% *vs.* 16%), *P* = 0.004. There was no carry-over/period effect. Expected adverse events were comparable in both groups, and no serious adverse events were reported.

**CONCLUSION:**

Atorvastatin reduced T-cell immune activation and exhaustion among cART-treated adults in a Ugandan cohort. Atorvastatin adjunct therapy should be explored as a strategy to improve HIV treatment outcomes among people living with HIV in sub-Saharan Africa.

## Introduction

The HIV/AIDS pandemic remains a major challenge to global health with 34 million people living with HIV (PLHIV) worldwide. The sub-Saharan Africa (SSA) region accounts for 69% of all PLHIV, 68% of new HIV infections among adults and 72% of the world’s AIDS-related deaths (UNAIDS 2012). Access to life-saving combination antiretroviral therapy (cART) has increased exponentially in the last 5 years, and up to 6 million people are receiving cART (WHO/UNAIDS/UNICEF 2011; UNAIDS 2012). With increasing numbers of PLHIV receiving cART for long periods, there is a need to optimise effectiveness of cART.

Evidence suggests that up to 40% of individuals exhibit suboptimal immune recovery during cART despite sustained viral suppression (Nakanjako *et al.* 2008). Among other factors, the immune activation associated with chronic HIV infection interferes with immunological recovery during suppressive cART (Hunt *et al.* 2011; Nakanjako *et al.* 2011). Our team previously reported persistent T-cell activation and exhaustion associated with suboptimal immune recovery after 4 years of suppressive cART (Nakanjako *et al.* 2011). Among Ugandan HIV-infected adults starting cART at CD4 < 200 cells/μl and achieving a plasma HIV RNA load <400 copies/ml, pre-cART immune activation independently predicted increased mortality (Hunt *et al.* 2011). Chronic persistent immune activation contributes to CD4 depletion in both untreated and successfully treated patients and increases the risk of non-AIDS-defining illnesses (NADIS), such as chronic kidney and coronary artery disease among PLHIV (Hunt *et al.* 2003, 2008; Gupta *et al.* 2009; Crowe *et al.* 2010). Therefore, novel therapeutic strategies aimed at preventing or reversing immune activation during cART are needed to maximise immune recovery and reduce HIV-associated morbidity and mortality among PLHIV in Africa.

Atorvastatin is a 3-hydroxy-3-methylglutaryl-coenzyme A reductase inhibitor, licensed and widely marketed for treatment of dyslipidaemia. However, a recent trial in the USA showed that short-term daily use of high-dose atorvastatin caused significant reductions in T-cell activation among HAART-naive, viremic, HIV-infected individuals (Ganesan *et al.* 2011). We therefore hypothesised that atorvastatin (80 mg daily for 12 weeks) could reduce immune activation levels among cART-treated suboptimal immune responders (SO-IR). In a randomised crossover placebo-controlled trial, we determined the anti-immune activation effects of 12 weeks of atorvastatin 80 mg daily *vs.* placebo, among SO-IR after 7 years of suppressive cART.

## Methods

### Study design

**Figure 1:**
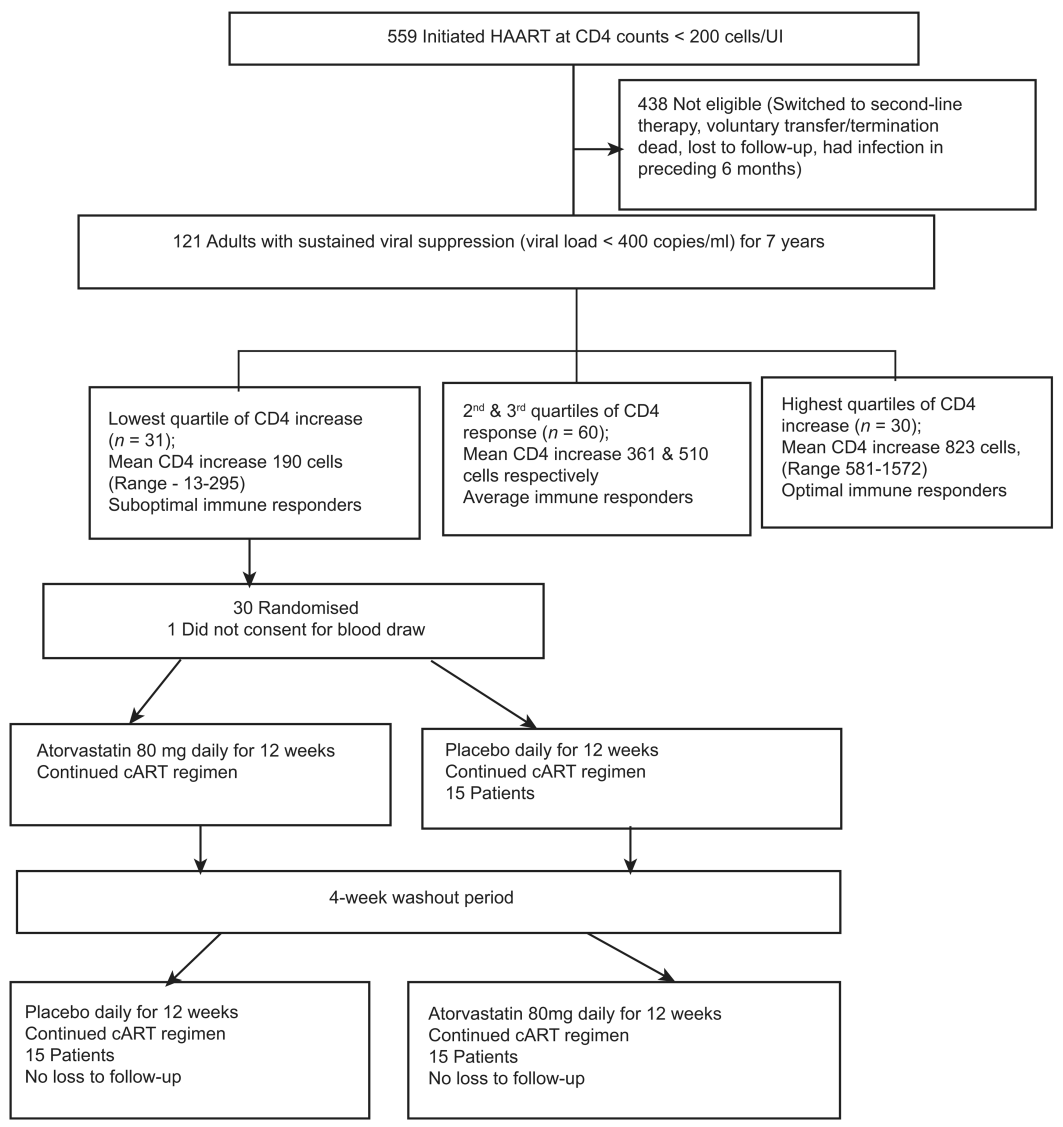
Profile of CD4 increase among 121 adults with sustained viral suppression for 7 years in the Infectious Diseases Institute research cohort.

In a randomised double-blind crossover placebo-controlled trial, individuals with SO-IR (patients with CD4 increase <295 cells after 7 years of suppressive cART) were randomised to atorvastatin, 80 mg daily or placebo for 12 weeks (phase 1). Upon completion of phase 1, participants had a 4-week washout period, subsequently switching treatment assignments to complete an additional 12 weeks (phase 2) in the opposite assignment (Figure 1).

### Study setting

This clinical trial was nested within the Infectious Diseases Institute (IDI) research cohort of adults that have received cART for at least 7 years. Between April 2004 and April 2005, 559 consecutive cART-naïve HIV-infected patients were initiated on cART and enrolled into the IDI prospective observational research cohort as previously described (Kamya & Mayanja-Kizza 2007). Patients were initiated on first-line cART at CD4 counts ≤200 cells/μl according to Ugandan guidelines for cART initiation at the time. Drugs were provided through the Global Fund [a generic combined formulation of stavudine (d4T), lamivudine (3TC) and nevirapine (NVP)] and the US President’s Emergency Plan for AIDS Relief [a combined formulation of zidovudine (ZDV) and 3TC plus efavirenz (EFZ) or nevirapine (NVP)]. Patients with toxicity to ZDV were changed to tenofovir (TDF). All patients received cotrimoxazole (or dapsone) prophylaxis according to the national policy to provide cotrimoxazole to all PLHIV. Adherence to cART was encouraged by at least three individual and group counselling sessions. Patients were reviewed monthly by the study physicians, who evaluated adherence to medication, toxicities and acute infections. HIV RNA viral loads, complete blood counts and CD4 lymphocyte counts were measured at 6-monthly intervals.

### Study population

After 7 years of cART, 121 patients were still receiving first-line cART and had sustained viral suppression (HIV RNA levels < 400 copies/ml) from 6 months post-cART initiation. The CD4 increase from baseline (at cART initiation) to current CD4 (at 7 years) was calculated and grouped into quartiles. Patients in the lowest quartile of CD4 increase with mean CD4 increase of 190 (minimum–maximum, −13 to 295) cells were grouped as ‘sub-optimal immune responders (SO-IR)’, *n* = 31, while patients in the highest quartile of CD4 increase were grouped as ‘optimal responders’, *n* = 30 with a mean CD4 increase of 823 (minimum–maximum, 581–1572) cells (Figure 1). We included 30 healthy HIV-negative, age- and gender-matched individuals consecutively selected from the HIV-negative patient register at Mulago routine HIV testing programme that is adjacent to the infectious diseases clinic, as a comparative group of immune activation in our healthy HIV-negative population. Ethical approval was provided by the Uganda National Council for Science and Technology, the national research ethical regulatory body and the National Drug Authority. The study was conducted according to the Declaration of Helsinki. All participants provided written informed consent to participate in the study. The clinical trial was registered by the Pan African Clinical Trial Registry at www.pactr.org (PACTR201301000445375). This trial was monitored by independent medical monitors under the IDI clinical trials unit, and a data safety monitoring board to ensure patient safety and protocol adherence.

We included HIV-infected adults receiving cART for at least 7 years, with a current CD4 < 295 cells/μl despite sustained viral suppression. We excluded pregnant women, individuals with a history of myositis, abnormal liver function tests, history of ingestion of lipid-lowering agents or use of therapeutic agents known to have substantial drug–drug interactions with statins such as protease inhibitors (PI)-containing cART. PIs increase atorvastatin area under the curve by 5.9 fold and may increase statin-induced toxicity (Alluvia Product characteristics). We also excluded individuals with a current opportunistic infection or febrile illness to avoid interference with our primary outcome of immune activation.

### Randomisation and blinding

Combination antiretroviral therapy-treated SO-IR were randomised to atorvastatin [80 mg (2 tablets) daily for 12 weeks] and placebo (two tablets daily), as adjunct therapy. Both placebo and atorvastatin were identical in appearance, supplied by Pfizer under investigator initiated research (Pfizer Reference # WI171060). Assignment of the drug and placebo, using computer-generated sequences, was completed by a pharmacist who was not involved with the clinical conduct of the study. Study clinicians were blinded to drug assignments with patient arm denoted as drug A and drug B. Patients’ clinical care results were reviewed in real time by an independent IDI research cohort doctor (not participating in the atorvastatin trial) as part of routine follow-up of patients in HIV treatment cohort.

### Patient follow-up

Patients had scheduled follow-up visits weekly in the first 4 weeks, and thereafter bi-weekly until completion of a study phase. Unscheduled patient visits were encouraged for any illnesses. Each scheduled clinical visit included clinical assessment for any expected/unexpected illnesses and adherence monitoring by self-report and pill counts for both antiretroviral drugs and study drug. Ongoing counselling and adherence support was provided by the study counsellor.

### Laboratory procedures

Patients had blood draws at initiation of the study drug, at weeks 12, 16 and 28. At each blood draw, complete blood counts, liver function tests and fasting serum lipid profile were measured at MBN Clinical laboratory, Kampala, Uganda within 2 h of sample collection.

### Peripheral blood mononuclear cells separation

Forty millilitres of whole blood, collected in acid citrate dextrose solution A vacutainers (BD VacutainerTM), were processed for Peripheral blood mononuclear cell (PBMC) separation within 2 h of collection. PBMCs were separated by Ficoll–Hypaque density configuration, washed and resuspended in phosphate buffer saline (PBS) containing heat-inactivated foetal calf serum (FCS). PBMCs were frozen and stored in FCS with 10% dimethyl sulfoxide (DMSO), in liquid nitrogen until assay time.

### Immunophenotyping

**Figure 2:**
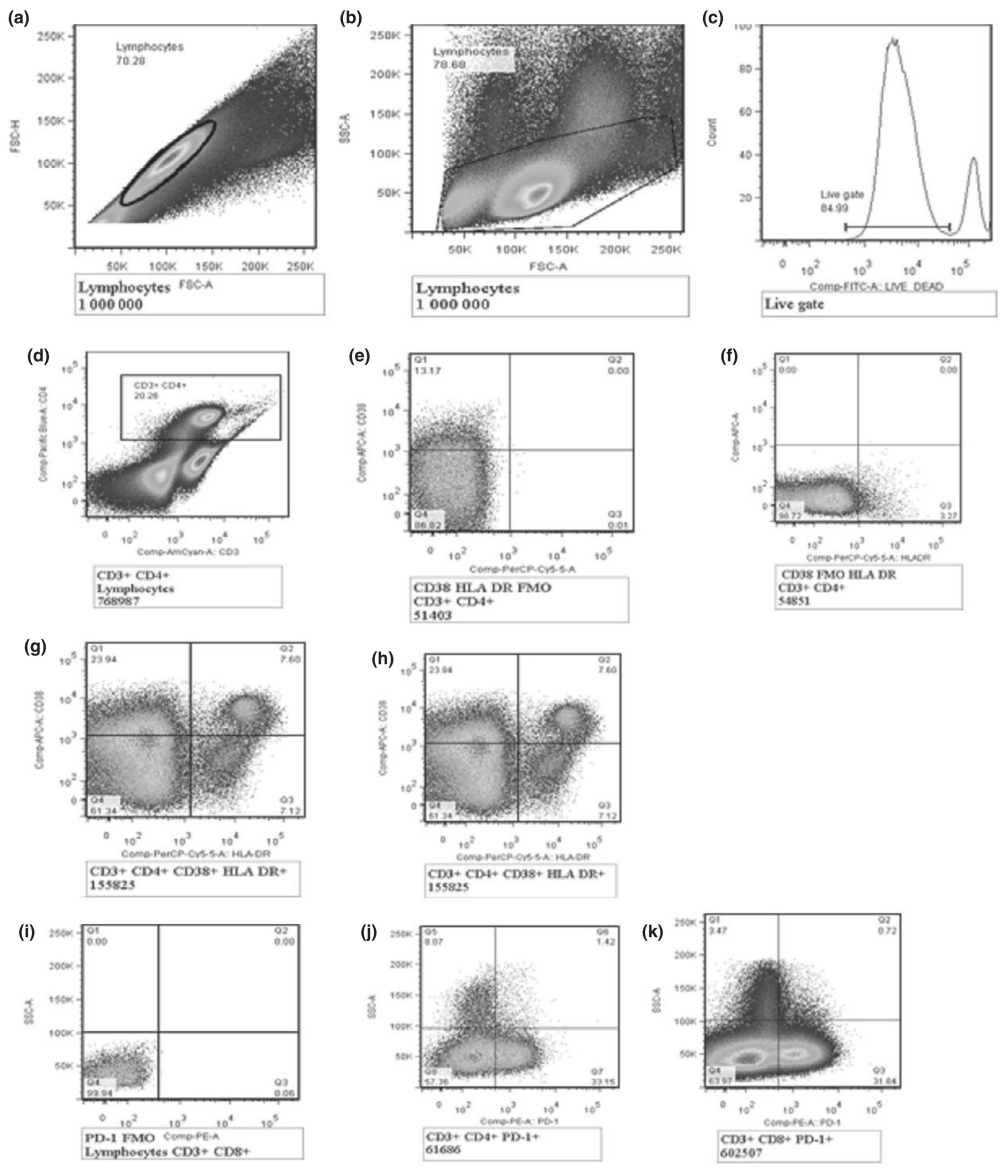
Gating strategy for T-cell activation and exhaustion. (a) shows singlets/duplets, (b) shows FSC with lymphocyte gate, (c) shows the live gate, (d) shows CD3 + CD4 + T cells, (e) shows CD38-APC *vs.* fluorescence minus one (FMO) HLADR-PerCP cy5.5, (f) shows FMO CD38-APC *vs.* HLADR- PerCP cy5.5, (g) shows CD4 T-cell activation (CD3 + CD4 + CD38 + HLADR+) and (h) shows CD8 T-cell activation (CD3 + CD8 + CD38 + HLADR+), (i) shows programmed death (PD-1) FMO, (j) shows CD4 T-cell exhaustion (CD3 + CD4 + PD1 + ) and (k) shows CD8 T-cell exhaustion (CD3 + CD8 PD1 + ).

Cryopreserved PBMC was thawed and batch analysed to ensure consistency, using an LSRII flow cytometer (BD Biosciences) at Uganda Virus Research Institute/Medical Research Council basic science laboratory. Cell surface staining was used to phenotype the markers for T-cell activation and exhaustion using antibodies for CD3- Amcyan, CD4- Pacific blue, HLADR-PerCP cy5.5, CD38-APC, PD-1-PE and Live/dead marker-FITC (BD Biosciences). Overall, at least 500 000 events in the CD3-positive gate were collected. The gating was standardised and set using fluorescence minus one controls for HLADR, CD38 and PD1 as shown in Figure 2. Percentages of activated T cells were determined by the proportion of CD3 + CD4 + (CD8) CD38 + HLADR+ T cells, and immune exhaustion was determined by the percentage of T cells expressing programmed cell death marker-1 (CD3 + CD4 + (CD8) PD-1) as shown in Figure 2.

### Primary outcome

The primary outcome of the trial was the change in CD4 T-cell activation levels during the 12-week intervention, in view of poor CD4 T-cell function observed among the suboptimal immune responders with persistently high immune activation levels despite suppressive antiretroviral therapy (Nakanjako *et al.* 2011, 2013). Change in CD8 T-cell activation and change in immune exhaustion markers were secondary outcomes.

### Statistical analysis

With a sample size of 30 (15 in each arm), this study had 80% power to detect a 0.25 difference in reduction of immune activation in the atorvastatin and placebo arm, with a statistical significance at *P*-value < 0.05. To assess the effects of treatment, we calculated the difference in changes, for immune activation and exhaustion parameters, while a participant was receiving atorvastatin or placebo. Pre- and post-treatment T-cell activation, percentage of activated T cells (CD3 + CD4 + (CD8)CD38 + HLADR+ cells) and exhaustion (CD3 + CD4 + (CD8)PD1 + ) were compared among SO-IR on atorvastatin (ATV) and placebo using the Mann–Whitney test for non-parametric tests. Additional analysis was carried out to determine carry-over and period effects using ANOVA test and the pk cross-STATA command. Given that there were no significant carry-over and period effects, results were presented for all the 30 participants for 12 weeks on atorvastatin *vs.* placebo. In addition, post-treatment immune activation and exhaustion were compared with the parameters among healthy donors from the same environment. Flow cytometry data were analysed using FlowJO software version and comparisons analysed using Prism Graph Pad 5.0 software and STATA version 11.0.

## Results

Demographic and clinical characteristics, including cART drug regimen, hypertension and biometric profile, haematology and liver function, were comparable among individuals randomised to atorvastatin and placebo (Table 1).

### T-cell activation markers

**Figure 3:**
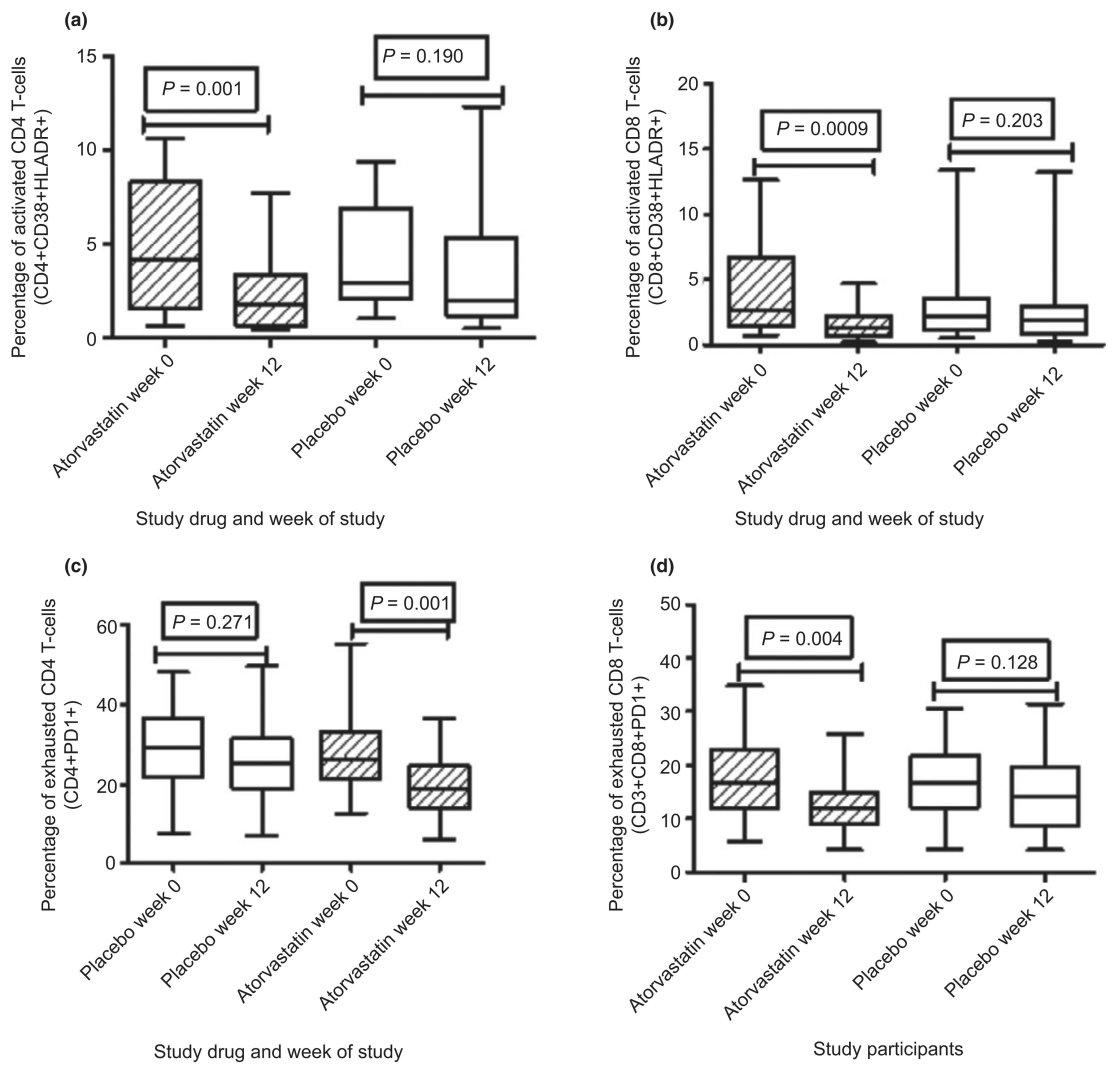
T-cell activation among cART-treated suboptimal immune responders before and after use of atorvastatin 80 mg daily *vs.* placebo. All 30 participants are presented for the 12 weeks on study drug (atorvastatin) *vs.* placebo. (a) Shows percentages of activated CD4 T cells, (b) shows percentages of activated CD8 T cells, (c) shows percentages of exhausted CD4 T cells and (d) shows percentages of exhausted CD8 T cells. The whiskers represent the interquartile ranges. The non-parametric Mann–Whitney test was used to compare immune activation in the study groups, with statistical significance at *P*-value ≤ 0.05.

Twelve weeks of atorvastatin adjunct therapy to cART showed a 28% higher reduction in percentage of activated CD4 T cells than placebo and cART; median percentage of activated CD4 T cells [Interquartile range (IQR), from 4.2 (3.7–6.2) to 1.7 (1.6–3.1) in the atorvastatin arm, *P* = 0.001 and from 2.9 (2.9–5.0) to 2.0 (2.3–4.9) in the placebo arm, *P* = 0.190]. A 12-week atorvastatin and cART also led to a 35% higher reduction in percentage of activated CD8 T cells than cART and placebo, from 2.6 (2.5–5.0) to 1.3 (1.1–1.9) in the atorvastatin arm, *P* = 0.0009 and from 2.1 (2.0–4.7) to 1.9 (1.4–3.3) in the placebo arm, *P* = 0.203 (Figure 3). There was no carry-over (*P* = 0.539) or period effect (*P* = 0.865).

### T-cell exhaustion

Twelve weeks of atorvastatin adjunct therapy to cART showed a 14% higher reduction in the percentage of exhausted CD4 T cells (CD3 + CD4 + PD1 + ) than placebo; from 26.0 (24.5–32.5) to 19.0 (16.9–22.6) in the atorvastatin arm, *P* = 0.004 and from 29.2 (25.4–32.5) to 25.3 (22.2–30.2) in the placebo arm, *P* = 0.271. In addition, 12 weeks of cART and atorvastatin showed an 11% higher reduction in percentage of exhausted CD8 T cells from 16.7 (14.9–20.5) to 12.1 (10.5–14.8) in the atorvastatin arm, *P* = 0.004 and from 16.9 (14.9–20.0) to 14.2 (12.3–17.9) in the placebo arm, *P* = 0.128 (Figure 3). Post-therapy T-cell activation and exhaustion (for both atorvastatin and placebo) were significantly higher than levels among healthy HIV-negative individuals from the same community (data not shown). The smallest reductions in immune activation were noted among patients receiving an efavirenz-containing cART regimen while the four highest reductions in immune activation were noted among patients receiving a nevirapine-containing cART regimen.

### Safety and tolerability

Atorvastatin (80 mg daily) was well tolerated as adjunct to nevirapine and efavirenz-containing first-line cART. Liver function tests were comparable among HIV-infected adults receiving cART plus atorvastatin and individuals receiving cART and placebo (Table 2).

### Lipid profile

Low-density lipoprotein (LDL) and triglycerides reduced significantly among individuals receiving atorvastatin after 12 weeks of therapy in phase 1 (*P*-value 0.0006) and phase 2 (*P*-value 0.014) (Table 2).

### Adverse events

Expected adverse events were comparable among individuals on atorvastatin (12) and placebo (9), with myalgias as the commonest in six individuals (three in atorvastatin and three in placebo arms), followed by chest pain in four individuals (three in atorvastatin and one in placebo arms), headache in four individuals (one in atorvastatin and three in placebo arms) and arthralgia in three individuals (two in atorvastatin and one in placebo arm). Other expected adverse events included backache (1), neck pain (1), left forearm pain (1); only reported in the atorvastatin arm). All adverse events resolved spontaneously without unblinding of study participants.

## Discussion

We found that short-term adjunct therapy with high-dose atorvastatin decreased CD4 and CD8 activation and exhaustion among cART-treated adults with 7 years of suppressive cART in an African cohort. To our knowledge, this is the first study to report use of atorvastatin as adjunct to cART in SSA, where individuals reportedly have high background immune activation due to several endemic infections such as malaria, intestinal helminths and tuberculosis (Borkow *et al.* 2001; Coban *et al.* 2005).

Our results are consistent with a previous clinical trial in the USA where high-dose atorvastatin (80 mg daily) was reported to decrease CD4 and CD8 T-cell activation among cART-naive HIV-infected adults, as an incidental finding, although atorvastatin did not demonstrate antiretroviral properties (Ganesan *et al.* 2011). Similarly, previous studies in USA and Europe reported anti-inflammatory effects of statins during HIV infection (Ono & Freed 2001; del Real *et al.* 2004; Giguere & Tremblay 2004) and in management of atherosclerosis (Jain & Ridker 2005). Statins have been widely prescribed for their cholesterol-lowering properties and efficacy in cardiovascular disease; however, compelling evidence now exists that statins also have extensive immunomodulatory properties that operate independently (Greenwood *et al.* 2006). Statins also act as direct inhibitors of induction of MHC-II expression by IFN-gamma and thus as repressors of MHC-II-mediated T-cell activation, through inhibition of the inducible promoter IV of the transactivator CIITA in several cell types, including primary human endothelial cells and monocyte–macrophages (Kwak *et al.* 2000).

In view of previous reports that T-cell immune activation and exhaustion were associated with poor immune recovery and mortality during antiretroviral therapy (Nakanjako *et al.* 2008, 2011; Hunt *et al.* 2011), our current report of anti-immune activation effects of atorvastatin presents an opportunity for utilisation of atorvastatin and probably other statins to optimise immune recovery among cART-treated HIV-infected individuals in Africa. In addition, use of atorvastatin to reduce immune activation could be explored in the management of the immune reconstitution inflammatory syndrome (IRIS), which is reportedly associated with high levels of immune activation (Bonham *et al.* 2008; Goovaerts *et al.* 2013). Furthermore, with the evidence that persistent immune activation during chronic HIV infection was associated with increased risk of non-AIDS illnesses (NADIS) such as atherosclerosis and kidney diseases (Gupta *et al.* 2009; Crowe *et al.* 2010; Kaplan *et al.* 2011), there are potential benefits of atorvastatin therapy in the reduction of NADIS among adults ageing with HIV. It is important to note that atorvastatin demonstrated the conventional lipid-lowering effects in our study participants, consistent with previous reports of reduced cardiovascular risk associated with use of atorvastatin (Ray & Cannon 2005; Acharjee & Welty 2008). Therefore, atorvastatin adjunct therapy could benefit cART-treated individuals through its reduction of persistent immune activation associated with chronic HIV infection (Hunt *et al.* 2011; Nakanjako *et al.* 2011) and through its known lipid-lowering effects among cART-treated adults at risk of atherosclerosis (Ssinabulya *et al.* 2014).

However, we observed that effects of atorvastatin were not carried over through the washout period and the effects were independent of the sequence of interventions. Our study implies that HIV-infected cART-treated individuals might benefit from long term rather than short-term use of atorvastatin. This finding is similar to previous reports of improved clinical outcomes among elderly individuals receiving long-term atorvastatin (Ray & Cannon 2005; Acharjee & Welty 2008). One unexplained finding is the observed rise in immune activation during the washout period in both study arms, although relatively higher among the individuals that started with atorvastatin. We postulate that it could be due to environmental variation of other possible causes of immune activation that were not evaluated in our study. However, we believe that such variation did not interfere with interpretation of our data given the randomised crossover study design.

### Safety and tolerability of atorvastatin during antiretroviral therapy

We used the highest FDA-approved dose of atorvastatin for maximal lipid-lowering effects previously used among cART-naive HIV-infected adults (Ganesan *et al.* 2011). High-dose atorvastatin was well tolerated as adjunct therapy to nevirapine and efavirenz-based cART regimen. Our findings are consistent with recent clinical trials that demonstrated minimal adverse events with high statin doses, such as 0.6% serious hepatic and 1.3% musculo-skeletal adverse effects (Archbold & Timmis 1998; LaRosa *et al.* 2005). In addition, the Myocardial Ischaemia Reduction with Aggressive Cholesterol Lowering (MIRACL), Treating to New Targets (TNT), Pravastatin or Atorvastatin Evaluation and Infection Therapy – Thrombolysis in Myocardial Infarction 22 (PROVE ITTIMI 22) and Stroke Prevention by Aggressive Reduction in Cholesterol Levels (SPARCL) trials, have shown that high-dose statins, especially atorvastatin 80 mg, can reduce vascular risks more than low or moderate-dose statin therapy (Archbold & Timmis 1998; LaRosa *et al.* 2005). Pharmacokinetics trials previously showed that efavirenz reduced atorvastatin exposure by 43% and the total active atorvastatin by 34% (Gerber *et al.* 2005). Although we did not conduct pharmacokinetic studies, the four least reductions in immune activation were recorded among patients receiving an efavirenz-containing regimen. It is therefore likely that the reduced inhibition of 3-hydroxy-3-methylglutaryl-coenzyme A (HMG-CoA) reductase activity during co-administration of efavirenz may have resulted in diminished therapeutic effects of atorvastatin. In the management of acute coronary syndromes, atorvastatin 80 mg was well tolerated and resulted in better clinical outcomes than standard care with pravastatin 40 mg (Ray *et al.* 2005). Given the known benefits of high-dose atorvastatin therapy, our study implies additional benefit of adjunct atorvastatin therapy among HIV-infected adults receiving antiretroviral therapy. We therefore recommend further studies to determine the optimal dosing of atorvastatin, as adjunct therapy to non-nucleoside reverse transcriptase inhibitor (NNRTI)-containing cART.

A major strength of our study was the randomised double-blinded crossover design that controlled for confounders of individuals’ immune activation levels (Sibbald & Roberts 1998; Valentinuzzi 2004). Twelve weeks’ administration of atorvastatin did not show significant effect on absolute CD4 count (data not shown), probably because of the short duration of therapy. Therefore, further research is required to determine long-term immunological benefits of high-dose atorvastatin adjunct therapy. We did not evaluate other causes of immune activation in this environment that could have accounted for the increasing levels of immune activation during the wash-out period. However, post-treatment immune activation levels remained significantly higher than immune activation levels among age- and gender-matched healthy HIV-negative individuals selected from the same setting. Nevertheless, our proof of concept that atorvastatin adjunct therapy provides additional reduction of immune activation may inform the development of larger and longer studies to evaluate the effect of atorvastatin adjunct therapy on HIV treatment outcomes.

## Conclusion

Atorvastatin reduced T-cell immune activation and exhaustion among cART-treated adults with suboptimal immune recovery in an urban Ugandan cohort. Atorvastatin adjunct therapy should be explored as a strategy to improve HIV treatment outcomes among cART-treated adults with persistently high levels of immune activation.

## Figures and Tables

**Table 1:** Baseline characteristics of HAART-treated HIV-infected adults with suboptimal immune response within the IDI research cohort

Variables	Active drug first (*N* = 15)	Placebo first (*N* = 15)
Demographic data		
Female *n* (%)	7 (46.7)	10 (66.7)
Median age (IQR)	41 (40–50)	47 (43–51)
Marital status *n* (%)		
Married/cohabiting	10 (66.7)	6 (40.0)
Divorced	3 (20.0)	4 (26.7)
Widowed	2 (13.3)	5 (33.3)
Occupation *n* (%)		
Unemployed	4 (26.7)	2 (13.3)
Employed	11 (73.3)	13 (86.7)
Education *n* (%)		
Primary	6 (40.0)	6 (40.0)
Secondary and above	9 (60.0)	9 (60.0)
CD4 at HAART initiation, median (IQR)	78 (43–162)	92 (50–130)
First-line HAART regimen, *n* (%)		
ZDV+3TC+NVP	5 (33.3)	7 (46.7)
ZDV+3TC+EFZ	5 (33.3)	3 (20.0)
TDF+FTC+EFZ	1 (6.7)	0 (0.0)
TDF+FTC+NVP	1 (6.7)	0 (0.0)
Others	2 (13.3)	3 (20.0)
Concurrent medication*		
Antihypertensive *n* (%)	0 (0.0)	3 (20)
Cotrimoxazole prophylaxis	15 (100)	14 (87)
Fluconazole	0 (0)	1 (7)
Physical examination		
Axillary temperature, median (IQR)	36.0 (35.7–36.4)	36.1 (35.8–36.4)
BMI, median (IQR)	20.8 (19.5–21.6)	24.0 (21.1–26.4)
Waist circumference, median (IQR)	79.0 (72.0–80.0)	88.0 (77.0–96.0)
Hip circumference, median (IQR)	88.0 (86.0–95.0)	97.0 (93.0–104.0)
Median waist to hip ratio(IQR)	0.9 (0.8–0.9)	0.9 (0.9–1.0)
Median pulse rate (IQR)	80.0 (70.0–92.0)	84.0 (77.0–94.0)
Systolic blood pressure, median (IQR)	110 (100–149)	140 (112– 163)
Diastolic blood pressure, median (IQR)	74 (65–82)	89 (70–100)
HB (g/dl), median (IQR)	14 (12.9–15.2)	13.1 (12.2–14.2)
WBC (10^3^ cells/Ul), median (IQR)	3.6 (2.8–3.8)	3.45 (3.0–4.1)
ESR (mm/h), median (IQR)	11 (3–15)	11 (3–15)

* No participants had any history of medication with non-steroidal anti-inflammatory drugs (NSAIDS), steroids, use of statins, family history of stroke and sudden death, febrile illness or confirmed malaria within 2 weeks preceding the study.

WBC, White blood cell count, normal adult range, 4.0–11.0 × 10^3^ cells/Ul; HB, haemoglobin, normal range, 12–18 g/dl; ESR, erythrocyte sedimentation rate, normal range (male, female, elderly, up to 10, 15, 20 mm/h).

**Table 2:** Liver function tests and fasting lipid profile of participants by study arm

Laboratory parameter, Median (IQR)	Week	Placebo	Active drug	*P*-value†
AST (U/l)	0	35.8 (25.3, 55.9)	35.8 (24.5, 43.8)	0.458
	12	32.8 (25.9, 44.3)	32.6 (25.6, 44.6)	0.852
	16	34.6 (26.5, 45.9)	33.3 (26.9, 41.4)	0.561
	28	32.3 (23.7, 49.0)	32.1 (23.6, 45.4)	0.458
ALT(U/l)	0	18.1 (11.1, 32.9)	15.6 (11.1, 32.9)	0.694
	12	15.1 (9.1, 20.6)	13.3 (6.2, 17.6)	0.272
	16	16.0 (12.6, 26.3)	15.9 (10.9, 23.4)	0.319
	28	14.6 (7.2, 19.9)	11.9 (7.2, 21.1)	0.948
HDL (mmol/l)	0	1.7 (1.5, 2.0)	1.7 (1.6, 1.8)	0.726
	12	1.7 (1.5, 2.0)	1.5 (1.2, 1.9)	0.113
	16	1.9 (1.6, 2.5)	1.6 (1.6, 2.0)	0.492
	28	1.5 (1.3, 1.6)	1.5 (1.3, 1.8)	0.380
LDL (mmol/l)	0	4.9 (2.4, 6.7)	3.1 (2.2, 4.9)	0.222
	12	4.2 (2.1, 6.4)	1.0 (0.9, 2.5)	0.0006***
	16	3.8 (2.5, 6.4)	2.3 (1.9, 4.8)	0.097
	28	1.9 (1.3, 3.1)	2.1 (1.7, 5.1)	0.239
Triglycerides (mmol/l)	0	2.0 (1.4, 3.2)	1.6 (1.1, 2.4)	0.089
	12	1.7 (0.9, 2.2)	1.0 (0.7, 2.1)	0.014*
	16	2.6 (1.3, 3.5)	1.7 (1.2, 2.8)	0.178
	28	1.5 (1.2, 2.1)	1.5 (1.2, 2.3)	0.663

† Mann–Whitney test.

* *P* value < 0.05,

*** *P* value < 0.001

AST, Aspartate aminotransferase, normal range 0–40 μl; ALS, alanine aminotransferase, normal range 0–40 μl; HDL, high-density lipoprotein, normal range 1–1.8 mmol/l; LDL, low-density lipoprotein, normal range up to 4.4 mmol/l.
